# O-GlcNAc modified-TIP60/KAT5 is required for PCK1 deficiency-induced HCC metastasis

**DOI:** 10.1038/s41388-021-02058-z

**Published:** 2021-10-14

**Authors:** Rui Liu, Dongmei Gou, Jin Xiang, Xuanming Pan, Qingzhu Gao, Peng Zhou, Yi Liu, Jie Hu, Kai Wang, Ni Tang

**Affiliations:** Key Laboratory of Molecular Biology for Infectious Diseases (Ministry of Education), Institute for Viral Hepatitis, Department of Infectious Diseases, The Second Affiliated Hospital, Chongqing Medical University, 400010 Chongqing, China

**Keywords:** Liver cancer, Metastasis

## Abstract

Aberrant glucose metabolism and elevated O-linked β-*N*-acetylglucosamine modification (O-GlcNAcylation) are hallmarks of hepatocellular carcinoma (HCC). Loss of phosphoenolpyruvate carboxykinase 1 (PCK1), the major rate-limiting enzyme of hepatic gluconeogenesis, increases hexosamine biosynthetic pathway (HBP)-mediated protein O-GlcNAcylation in hepatoma cell and promotes cell growth and proliferation. However, whether PCK1 deficiency and hyper O-GlcNAcylation can induce HCC metastasis is largely unknown. Here, gain- and loss-of-function studies demonstrate that PCK1 suppresses HCC metastasis in vitro and in vivo. Specifically, lysine acetyltransferase 5 (KAT5), belonging to the MYST family of histone acetyltransferases (HAT), is highly modified by O-GlcNAcylation in PCK1 knockout hepatoma cells. Mechanistically, PCK1 depletion suppressed KAT5 ubiquitination by increasing its O-GlcNAcylation, thereby stabilizing KAT5. KAT5 O-GlcNAcylation epigenetically activates *TWIST1* expression via histone H4 acetylation, and enhances MMP9 and MMP14 expression via c-Myc acetylation, thus promoting epithelial-mesenchymal transition (EMT) in HCC. In addition, targeting HBP-mediated O-GlcNAcylation of KAT5 inhibits lung metastasis of HCC in hepatospecific *Pck1*-deletion mice. Collectively, our findings demonstrate that PCK1 depletion increases O-GlcNAcylation of KAT5, epigenetically induces *TWIST1* expression and promotes HCC metastasis, and link metabolic enzyme, post-translational modification (PTM) with epigenetic regulation.

## Introduction

Metabolic reprogramming is a hallmark of cancer cells and supports the bioenergetic and biosynthetic demands of rapid growth and proliferation [1]. Most tumor cells preferentially metabolize glucose via glycolysis to produce energy even in oxygen-rich conditions, also known as the Warburg effect [2]. Indeed, alteration in the glycolytic pathway activation, such as hexokinase 2 (HK2), pyruvate kinase M2 (PKM2) and phosphofructokinase 1 (PFK1) induce glycolysis and provide pro-survival cues to tumor cells [3]. Gluconeogenesis, the reverse pathway of glycolysis, that produces glucose from non-carbohydrate carbon substrates such as lactate and pyruvate, plays crucial roles in metabolic reprogramming and tumor growth [4]. Gluconeogenesis is mainly controlled by three key enzymes –Phosphoenolpyruvate carboxykinase (PEPCK), fructose-1,6-bisphosphatase, and glucose-6-phosphatase. Recently, fructose-bisphosphatase 1 (FBP1) has been reported to suppress epithelial-mesenchymal transition (EMT) in hepatocellular carcinoma (HCC), indicating a negative role of gluconeogenesis pathway in HCC metastasis [5]. The cytoplasmic isoform of PEPCK, also known as PCK1 (or PEPCK-C), catalyzes the oxaloacetate (OAA) to phosphoenolpyruvate (PEP). PCK1 is upregulated in human melanoma and colon carcinoma, and promoted tumor growth [6, 7]. However, PCK1 was strikingly downregulated in HCC, and PCK1 depletion inhibited apoptosis or induced cancer cell proliferation and HCC growth in vivo [8–11]. Metastasis is an important characteristic of cancer and accounts for ~90% of cancer-associated deaths. Nevertheless, the role of PCK1 in HCC metastasis remains unclear.

EMT is the most important characteristic of tumor metastatic cells. It is accompanied by downregulation of genes associated with epithelial phenotype, upregulation of genes associated with mesenchymal phenotype, and high expression of genes belonging to the matrix metalloproteinases (MMPs) family [12, 13]. Epigenetic regulation and post-translational modifications (PTMs) are important in regulating EMT program [14]. The O-linked β-*N*-acetylglucosamine (O-GlcNAc) modification (O-GlcNAcylation) occurs on serine or threonine residues of proteins catalyzed by O-GlcNAc transferase (OGT) and removed by O-GlcNAcase (OGA). Increasing evidences have indicated that an increase in O-GlcNAcylation facilitates the process of tumorigenesis and metastasis [15]. Our previous study has shown that PCK1 depletion increases global O-GlcNAcylation levels through oxaloacetate accumulation, de novo UTP synthesis and AMPK-GFAT1 axis inactivation promoting uridine diphosphate-N-acetylglucosamine (UDP-GlcNAc) biosynthesis in hepatoma cells [16]. Several reports showed that transcriptional factors including Snail1, EMT-related markers such as E-cadherin, and epigenetic modulators, including EZH2, HDAC1, and SIRT1 can be O-GlcNAc-modified and are associated with cancer metastasis [17–21].

Lysine acetyltransferase 5 (KAT5, originally named TIP60 (HIV-1 Tat Interactive Protein, 60 kDa) is the catalytic subunit of the NuA4 acetyltransferase complex and participates in diverse cellular processes including transcriptional regulation, cell survival and proliferation [22]. KAT5 acetylates both core histones H2A, H3, and H4, and many important non-histone proteins associated with tumorigenesis and metastasis including p53, NF-κB, c-Myc, and Twist [23–27]. Various PTMs of KAT5 have been reported, such as phosphorylation, SUMOylation and ubiquitination, that regulates the HAT activity or interaction with other proteins [28–30]. However, the function of O-GlcNAcylation in KAT5 is far from explored.

In this study, we explored the potential functions of PCK1 and O-GlcNAcylation in HCC metastasis. We found that KAT5 was O-GlcNAcylated by OGT in PCK1-deficient hepatoma cells, and O-GlcNAcylation of KAT5 enhanced its stability. Furthermore, our data provide the evidence that PCK1 depletion increases O-GlcNAcylation of KAT5 and promotes EMT and metastasis in HCC. Finally, blocking O-GlcNAcylation suppressed metastasis of HCC in liver-specific *Pck1*-deletion mice. Thus, our findings provide new insights into the potential treatment of metastatic HCC.

## Results

### PCK1 attenuates invasion and metastasis of hepatoma cells in vitro and in vivo

**Fig. 1:**
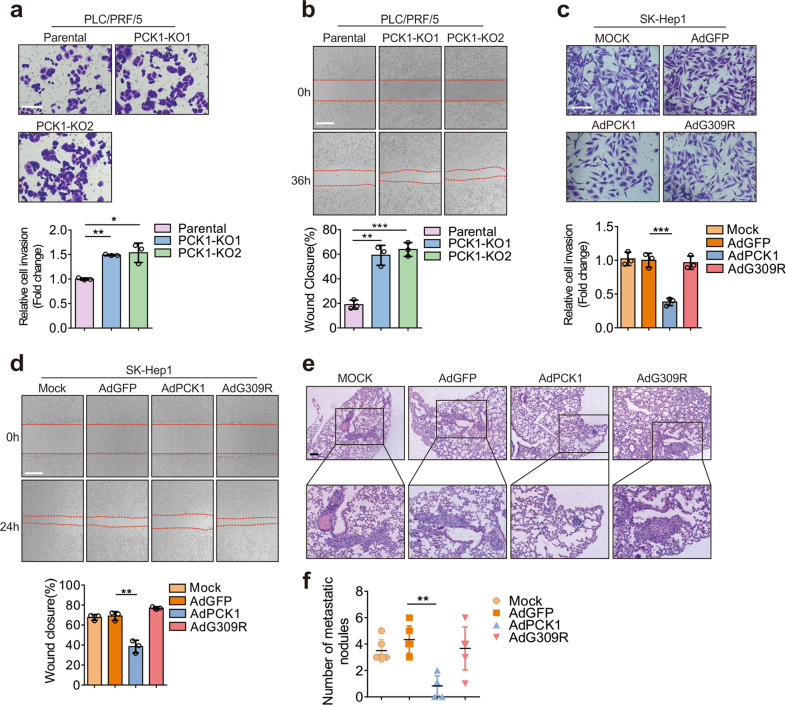
PCK1 suppresses hepatoma cell invasion and migration in vitro and in vivo. **a**–**d** Representative and quantified results of the transwell (**a**, **c**) and wound-healing assays (**b**, **d**) in parental or PCK1-KO PLC/PRF/5 cells (**a**, **b**) or in SK-Hep1 cells infected with GFP control (AdGFP), wild-type PCK1 (AdPCK1), or an enzymatic deficient mutant of PCK1 (AdG309R), mock is a blank control (**c**, **d**). Statistical analysis was shown as mean ± SD (*n* = 3). One-way ANOVA followed by the Tukey test, *P < 0.05, **P < 0.01, ***P < 0.001. Scale bar: 100 μm. **e**, **f** Representative staining and quantification of metastatic nodules (**e**) and metastatic numbers (**f**) in the lung of mice. MHCC-97H cells were infected with AdGFP, AdPCK1, or AdG309R and then injected into the left liver lobe of nude mice, mock is a blank control. Data was shown as mean ± SD (*n* = 6). One-way ANOVA followed by the Tukey test, **P < 0.01. Scale bar: 200 μm.

Cancer metastasis is an extremely complex process consisting of a series of sequential steps. The tumor cells possess properties of increased motility and invasiveness. To explore the role of PCK1 in hepatoma cell invasion and migration, we assessed the migratory potential of PCK1-knockout (PCK1-KO) PLC/PRF/5 cells as well as PCK1 overexpressing (PCK1-OE) SK-Hep1 cells. PCK1-KO greatly increased the invasion and migration abilities of PLC/ PRF/5 cells (Fig. 1a, b), whereas PCK1-OE significantly suppressed invasiveness of SK-Hep1 cells (Fig. 1c, d). Interestingly, the G309R mutant that causes enzyme deficiency of PCK1 [31] was unable to decrease the invasion and migration of SK-Hep1 cells (Fig. 1c, d), suggesting that the metabolic activity of PCK1 is required for suppressing the motility and invasiveness of hepatoma cells. To further explore the role of PCK1 in the hepatoma cell metastasis in vivo, MHCC-97H cells were infected with AdGFP, AdPCK1, or AdG309R recombinant adenoviruses and injected into the left lobes of nude mice livers. We found that wild-type (WT) PCK1, but not the G309R mutant, significantly suppressed lung colonization of hepatoma cells in nude mice, as determined by H&E staining of lung tissues (Fig. 1e) and metastatic nodules (Fig. 1f). Together, these results show that PCK1 suppresses invasion and metastasis of hepatoma cells in vitro and in vivo.

### PCK1 suppresses KAT5 O-GlcNAcylation

It has been demonstrated that enhanced O-GlcNAcylation plays important roles in HCC formation and progression [32]. We found that PCK1 downregulates global O-GlcNAcylation in hepatoma cells (Supplementary Fig. 1a, b), which is consistent with our previous study [16]. Importantly, we found that PCK1 inhibits motility of hepatoma cell via downregulating cellular O-GlcNAcylation levels by detecting the cellular motility in response to OGT or OGA inhibition (Supplementary Fig. 1c-j).

**Fig. 2:**
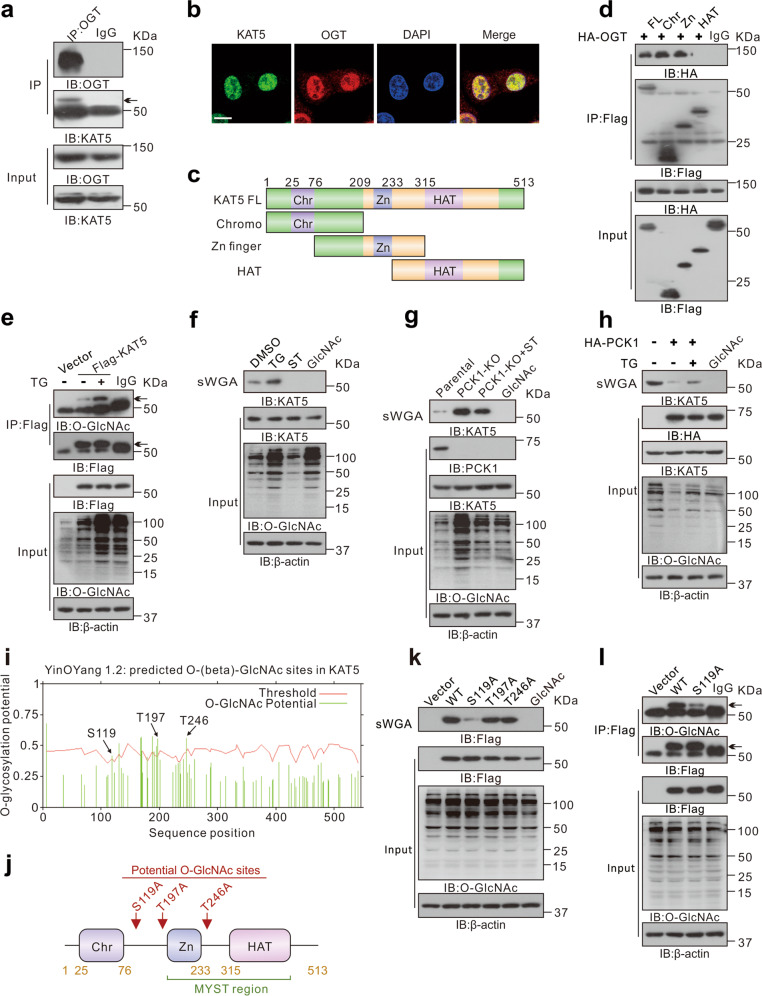
PCK1 depletion promotes O-GlcNAcylation of KAT5. **a** Interaction between endogenous OGT and KAT5 in MHCC-97H cells was detected by co-immunoprecipitation (co-IP) assay. **b** Subcellular co-localization of OGT and KAT5 in MHCC-97H cells were determined by immunofluorescence staining. Nuclei were counterstained with DAPI. Scale bar: 10 μm. **c**, **d** The interaction between HA-OGT and Flag-tagged full-length or truncated KAT5 (Chr 1-209aa, Zn 76-315aa, or HAT 233-513aa), as indicated in the diagram (**c**), were determined by co-IP in MHCC-97H cells (**d**). **e** Thiamet-G (TG) treatment enhances O-GlcNAc modification of KAT5. MHCC-97H cells were transfected with Flag-KAT5 or vector control for 48 h and treated with 25 μM TG for 12 h. Immunoprecipitation of Flag-KAT5 were performed with anti-FLAG antibody. **f** TG or ST045849 treatment regulates O-GlcNAc modification of KAT5. MHCC-97H cells were treated with 25 μM TG or 50 μM ST for 12 h, followed by succinylated wheat-germ agglutinin (sWGA) pull-down assay. Monosaccharide inhibitor GlcNAc (20 mM) was added as a negative control during sWGA-lectin-affinity purification. **g**, **h** PCK1-KO cells were treated with 50 μM ST for 12 h (**g**), MHCC-97H cells were transfected with vector control or HA-PCK1 for 48 h and treated with 25 μM TG for 12 h (**h**), followed by sWGA pull-down assay. **i** O-GlcNAc sites of KAT5 predicted using the YinOYang 1.2 server are shown with a black arrowhead at the top. The green vertical lines show the potential O-GlcNAc-modified Ser/Thr residues and the red horizontal wavy line indicates the threshold for modification potential. **j** Diagram of the potential O-GlcNAcylation sites on KAT5. **k**, **l** sWGA pull-down (**k**) or IP assay using anti-FLAG antibody (**l**). MHCC-97H cells were transfected with vector control, Flag-tagged WT, or mutants as indicated for 48 h, followed by sWGA pull-down or IP analysis using anti-FLAG antibody.

To further explore the mechanism by which OGT-mediated protein O-GlcNAcylation facilitates PCK1-KO cell migration and invasion, we screened for intracellular proteins that interact with OGT, using our previous immunoprecipitation assay coupled with mass spectrometry (IP-MS) data [16], and focused on lysine acetyltransferase 5 (KAT5), a histone acetyltransferase belonging to MYST family, that plays a significant role in tumor metastasis [27]. Endogenous or exogenous interactions between OGT and KAT5 were revealed using co-immunoprecipitation (Co-IP) experiments in MHCC-97H cells (Fig. 2a and Supplementary Fig. 1k, l). Confocal microscopy analysis verified that OGT and KAT5 were co-localized in the nucleus (Fig. 2b). To map the interaction region of KAT5 and OGT, we constructed serial truncated mutants of Flag-tagged KAT5. As shown in Fig. 2c, d, OGT interacted with the chromodomain and Zn-finger domain of KAT5. Then, we investigated the O-GlcNAc modification of KAT5 in hepatoma cells. Immunoprecipitated Flag-tagged KAT5 exhibited a distinct O-GlcNAc modification signal in MHCC-97H cells upon treatment with the OGA inhibitor Thiamet G (TG) (Fig. 2e). Furthermore, the succinylated wheat-germ agglutinin (sWGA) assay, a modified lectin that specifically binds to O-GlcNAc on proteins, was used to confirm endogenous KAT5 O-GlcNAcylation (Fig. 2f).

Next, we explored whether PCK1 regulated O-GlcNAcylation on KAT5. We found that PCK1 deficiency promoted KAT5 O-GlcNAcylation (Fig. 2g), but PCK1-OE displays an opposite effect (Fig. 2h). Furthermore, we predicted the potential site(s) of O-GlcNAcylation on KAT5 using the online database (YinOYang 1.2 Server). The results showed that Ser119, Thr197, and Thr246 had a high probability to be modified by O-GlcNAcylation (Fig. 2i, j). We demonstrated that S119A mutant markedly suppressed O-GlcNAcylation of KAT5 (Fig. 2k), which was further confirmed by immunoprecipitating Flag-tagged WT KAT5 or S119A mutant (Fig. 2l). Collectively, these data indicated that PCK1 decreases the levels of O-GlcNAcylation of KAT5.

### O-GlcNAcylation stabilizes KAT5 by inhibiting its ubiquitination

**Fig. 3:**
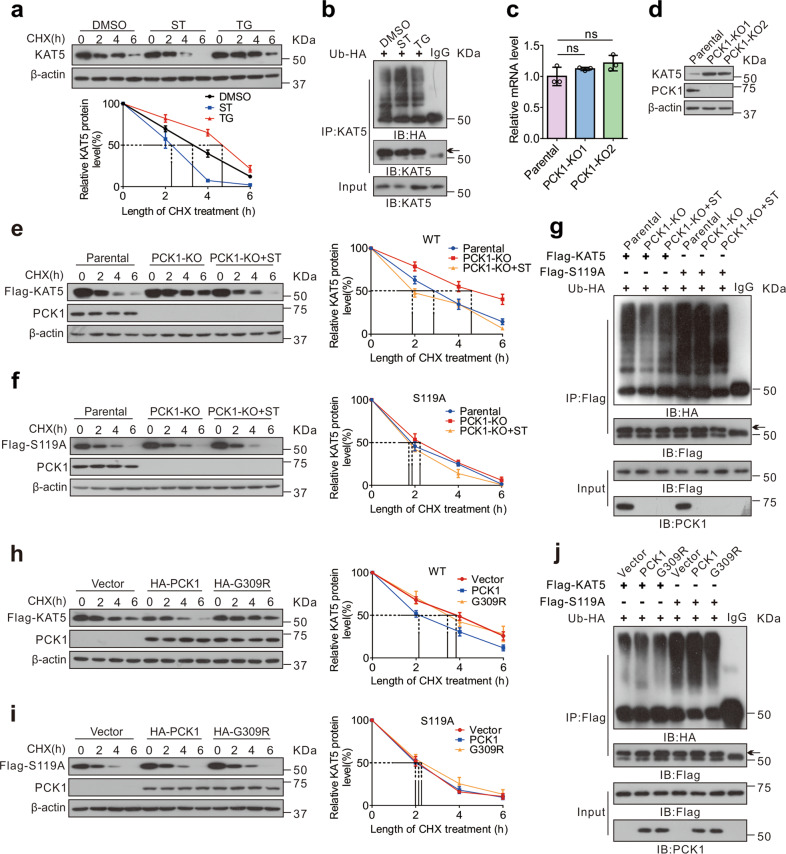
O-GlcNAcylation stabilizes KAT5 through suppression of its ubiquitination. **a** The half-life of endogenous KAT5 in MHCC-97H cells was measured by immunoblotting and quantitative analysis. Cells were treated with DMSO, 50 μM ST, or 25 μM TG for 12 h, and protein synthesis was blocked by treatment with 100 μM cycloheximide (CHX) for the indicated times. The levels of KAT5 were normalized to those of β-actin, and the 0 h points were arbitrarily set to 100%. Data are representative of at least three independent experiments. **b** Ubiquitination of endogenous KAT5 protein in MHCC-97H cells. Cells were transfected with HA-ubiquitin for 48 h and treated with DMSO, 50 μM ST or 25 μM TG for 12 h. Cell lysates were purified using anti-KAT5 antibody. Immunoprecipitated and input proteins were probed with the indicated antibodies. **c**, **d** The mRNA or protein expression levels of KAT5 in parental or PCK1-KO cells were measured by qRT-PCR (**c**) or immunoblotting (**d**). Statistical analysis was shown as mean ± SD (*n* = 3). ns not significant. **e**, **f** The half-life of Flag-tagged WT (**e**) or S119A mutant (**f**) in PCK1-KO cells treated with 50 μM ST was measured by immunoblotting and quantitative analysis. Protein synthesis was blocked by treatment with 100 μM CHX for the indicated times. The levels of KAT5 were normalized to those of β-actin, and the 0 h points were arbitrarily set to 100%. Data are representative of at least three independent experiments. **g** Ubiquitination of Flag-tagged WT or S119A mutant in PCK1-KO cells. **h**, **i** The half-life of Flag-tagged KAT5 WT (**h**) or S119A mutant (**i**) in MHCC-97H cells expressing PCK1 or G309R were measured by immunoblotting and quantitative analysis. The levels of KAT5 were normalized to those of β-actin, and the 0 h points were arbitrarily set to 100%. Data are representative of at least three independent experiments. **j** Ubiquitination of Flag-tagged WT or S119A mutant in MHCC-97H cells expressing PCK1 or G309R.

O-GlcNAc modification regulates protein function, protein–protein interactions, stability, localization, and enzyme activity [33]. The results showed that KAT5 was more stable, with a half-life of over 4 h, in MHCC-97H cells treated with Thiamet G, suggesting that KAT5 O-GlcNAcylation may enhance its stability and decrease its ubiquitination (Fig. 3a, b and Supplementary Fig. 2a, b). Interestingly, we detected that PCK1 has negligible effects on the expression levels of other KATs excluding KAT5 (Supplementary Fig. 2c, d). Meanwhile, we found that PCK1 reduced KAT5 protein expression without affecting its mRNA level (Fig. 3c, d and Supplementary Fig. 2e, f). Furthermore, compared with the parental cells, the half-life of KAT5 was prolonged and the ubiquitination of KAT5 was alleviated in PCK1-KO cells, while the S119A mutant or ST045849 (ST) treatment shortened the half-life of KAT5 and increased ubiquitination of KAT5 (Fig. 3e–g). Conversely, PCK1-OE reduced KAT5 stability and promoted KAT5 ubiquitination, but the G309R mutant had no effect (Fig. 3h–j). These data showed that PCK1 deletion stabilizes KAT5 by promoting KAT5 O-GlcNAcylation, thereby inhibiting its ubiquitination and degradation.

### PCK1 deficiency promotes EMT through epigenetic activation of *TWIST1* by O-GlcNAcylation of KAT5

**Fig. 4:**
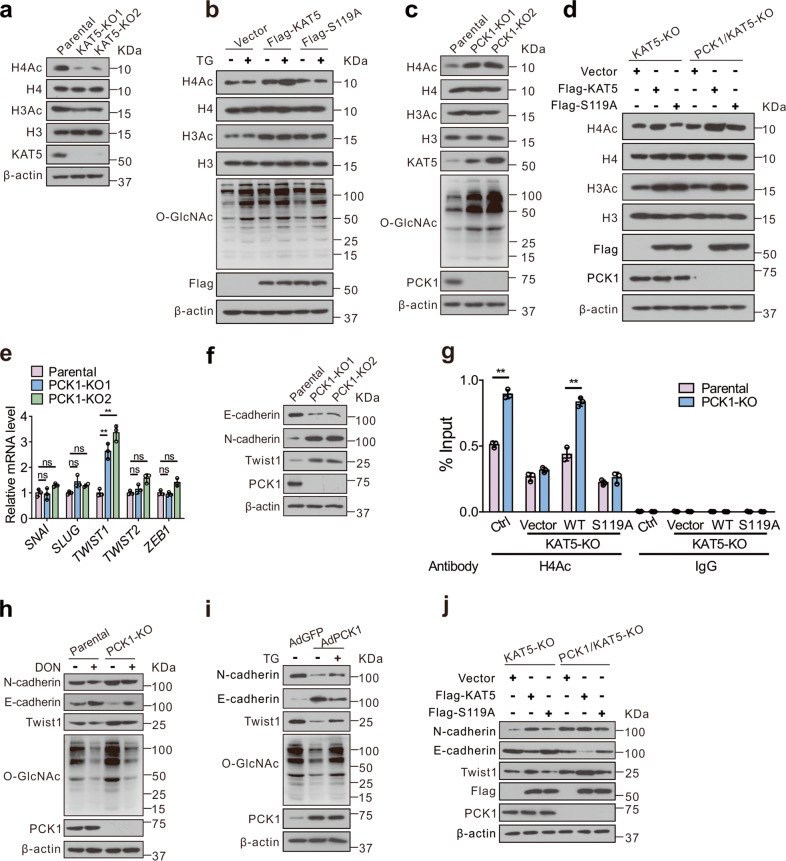
O-GlcNAcylation of KAT5 promotes H4Ac enrichment in *TWIST1* promoter and expression of EMT markers in PCK1-KO cells. **a**, **b** Histone H3 acetylation (H3Ac) and histone H4 acetylation (H4Ac) levels were determined by immunoblotting in parental or KAT5-KO cells (**a**) or in KAT5-KO cells transfected with vector control, Flag-tagged WT, or S119A mutant for 48 h and treated with 25 μM TG for 12 h (**b**). **c**, **d** H3Ac and H4Ac were determined. **e** The mRNA expression levels of EMT-related transcription factors were measured by qRT-PCR. Statistical analysis was shown as mean ± SD (*n* = 3). One-way ANOVA followed by the Tukey test, ns not significant, **P < 0.01. **f** The protein expression levels of EMT-related markers were measured in parental or PCK1-KO cells. **g** H4 acetylation levels in *TWIST1* gene promoter region were determined by chromatin immunoprecipitation (ChIP) assays. Cells were treated as indicated. IgG was used negative control. Statistical analysis was shown as mean ± SD (*n* = 3). Student’s *t* test, **P < 0.01. **h**–**j** The protein expression levels of EMT-related markers were measured by immunoblotting in PCK1-KO cells (**h**), PCK1-OE MHCC-97H cells (**i**), or KAT5-KO cells (**j**) treated as indicated.

Then, we investigated whether loss of PCK1-mediated acceleration of the migration and invasion of hepatoma cells is dependent on KAT5. We found that depletion of KAT5 reduced cell migration and invasion (Supplementary Fig. 3a, b). Since KAT5 acetylates both histone proteins including H2A, H3, and H4, and non-histone proteins, such as c-Myc [23, 26], we subsequently checked the levels of histone H3 acetylation (H3Ac) and histone H4 acetylation (H4Ac) in KAT5-KO cells. As expected, KAT5 depletion resulted in a dramatic decrease in H3Ac and H4Ac level (Fig. 4a). Expression of WT KAT5, but not S119A mutant, could markedly upregulate H4 acetylation by TG treatment. Interestingly, both WT and S119A mutant increased H3 acetylation (Fig. 4b) in KAT5-KO cells, indicating that H4 acetylation was specifically affected by O-GlcNAcylation of KAT5.

In addition, gain-and loss-of-function assays indicated that PCK1 regulates H4 acetylation, whereas H3 acetylation had no change (Fig. 4c and Supplementary Fig. 3c), suggesting a potential link between PCK1 and KAT5 O-GlcNAcylation on these epigenetic modifications. Furthermore, we found that WT KAT5 restored H4 acetylation notably under PCK1 depletion, whereas S119A mutant failed to rescue (Fig. 4d), suggesting that PCK1 deficiency increased H4 acetylation via KAT5 O-GlcNAcylation.

Given that histone acetylation is a ubiquitous hallmark of transcriptional activity [34], we performed qRT-PCR to identify the potential transcriptional targets possibly regulated by PCK1. *TWIST1* was upregulated in PCK1-KO cells (Fig. 4e), but downregulated in PCK1-OE MHCC-97H cells (Supplementary Fig. 3d). As a key transcriptional factor in EMT [12, 35], *TWIST1* maybe the downstream transcriptional target involved in PCK1-mediated suppression of HCC metastasis. To test this hypothesis, we examined the expression of Twist1, E-cadherin, N-cadherin in PCK1-KO cells and PCK1-OE MHCC-97H cells. The levels of Twist1, N-cadherin were upregulated but that of E-cadherin was downregulated in PCK1-KO cells, whereas PCK1-OE cells displayed opposite effects (Fig. 4f and Supplementary Fig. 3e). To verify whether PCK1 regulates *TWIST1* expression by KAT5 O-GlcNAcylation, we examined H4Ac levels on the *TWIST1* gene promoter region by Chromatin immunoprecipitation (ChIP) assay and found that WT KAT5 but not S119A mutant led to the increased level of H4Ac on *TWIST1* promoter and enhanced expression of *TWIST1* in PCK1-KO cells (Fig. 4g). Moreover, inhibition of O-GlcNAcylation using 6-diazo-5-oxo-L-norleucine (DON) or shOGT partially reversed the positive effects caused by PCK1 deficiency (Fig. 4h and Supplementary Fig. 3f). Conversely, PCK1-OE resulted in reversed regulatory effects on these molecules (Fig. 4i and Supplementary Fig. 3g). Furthermore, we found that WT KAT5, but not S119A mutant, partially offset the regulatory effects on Twist1, N-cadherin and E-cadherin expression mediated by PCK1 deficiency (Fig. 4j), suggesting KAT5 O-GlcNAcylation plays an essential role in transcriptional regulation of the expression of *TWIST1* and EMT markers. These data suggest PCK1 deficiency accelerate EMT process via epigenetic activation of *TWIST1* expression.

### Loss of PCK1 accelerates EMT process via upregulation of KAT5 O-GlcNAcylation and c-Myc acetylation

**Fig. 5:**
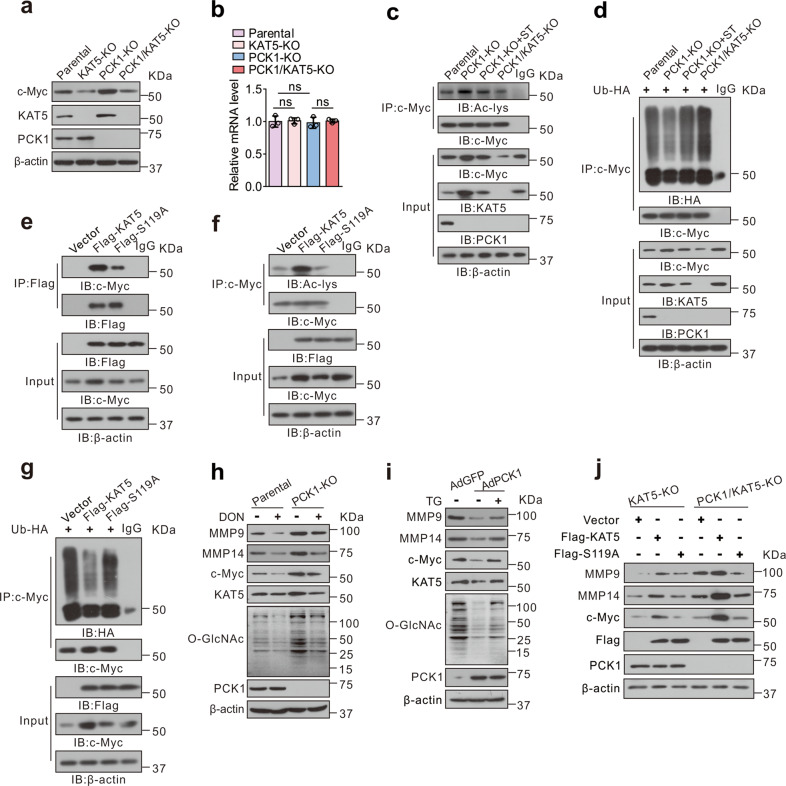
KAT5 O-GlcNAcylation enhances MMP9 and MMP14 expression through c-Myc acetylation in PCK1-KO cells. **a**, **b** The protein or mRNA expression levels of c-Myc in KAT5-KO or PCK1-KO cells. Statistical analysis was shown as mean ± SD (*n* = 3). One-way ANOVA followed by the Tukey test, ns not significant. **c**, **d** Acetylation (**c**) or ubiquitination (**d**) of c-Myc in parental or PCK1-KO cells. Cells were treated as indicated, followed by immunoprecipitation with anti-c-Myc antibody. **e** Interaction between KAT5 WT or S119A mutant and c-Myc in KAT5-KO cells was detected by co-immunoprecipitation (CoIP) assay. **f**, **g** Acetylation (**f**) or ubiquitination (**g**) of c-Myc in KAT5-KO cells. Cells were transfected with vector control, Flag-tagged WT, or S119A mutant for 48 h, followed by immunoprecipitation with anti-c-Myc antibody. **h**, **i** The protein expression levels of EMT-related markers were measured by immunoblotting in PCK1-KO cells (**h**), PCK1-OE MHCC-97H cells (**i**), or KAT5-KO cells (**j**) treated as indicated.

In addition to regulating the transcription of *TWIST1*, it has been reported that KAT5 increases the stability of c-Myc through acetylation of c-Myc, thereby promoting tumor invasion and metastasis [36]. We explored whether PCK1 inhibits EMT process by inhibiting KAT5 O-GlcNAcylation and c-Myc acetylation, and first found that PCK1-KO increased c-Myc protein levels, and KAT5-KO reduced c-Myc protein levels, although the c-Myc mRNA level was not changed (Fig. 5a, b). Then, we detected that PCK1 depletion promoted c-Myc acetylation and inhibited its ubiquitination. In contrast, ST treatment or KAT5-KO reduced c-Myc acetylation and accelerated its ubiquitination and degradation (Fig. 5c, d). Next, we investigated whether KAT5 O-GlcNAcylation affects its interaction with c-Myc. S119A, the O-GlcNAc-deficient mutant of KAT5, weakened the association with c-Myc (Fig. 5e). In addition, WT KAT5 markedly increased the acetylation of c-Myc and reduced its ubiquitination, whereas S119A mutant had lesser effect (Fig. 5f, g). These data indicated that PCK1 inhibits c-Myc acetylation by suppressing KAT5 O-GlcNAcylation.

Furthermore, PCK1 deficiency promoted KAT5, c-Myc and EMT-related marker MMP9, MMP14 expression. DON or shOGT partially offset the stimulating effects mediated by PCK1 deficiency (Fig. 5h and Supplementary Fig. 4a). Conversely, PCK1-OE resulted in reversed regulatory effects on these molecules (Fig. 5i and Supplementary Fig. 4b). We further examined whether KAT5 O-GlcNAcylation was responsible for cell invasion and migration. WT KAT5 increased c-Myc, MMP9, MMP14 expression and promoted cell invasion and migration, whereas the S119A mutant failed to exert this stimulatory role on tumor metastasis (Fig. 5j and Supplementary Fig. 4c, d), suggesting that O-GlcNAcylation plays an essential role in KAT5 activation, resulting in c-Myc accumulation and HCC invasive phenotype. Those data suggest PCK1 deficiency accelerates the EMT process by promoting KAT5 O-GlcNAcylation and c-Myc acetylation.

### Targeting O-GlcNAcylation reverses DEN/PB-induced HCC lung metastasis in liver-specific *Pck1*-deletion mice

**Fig. 6:**
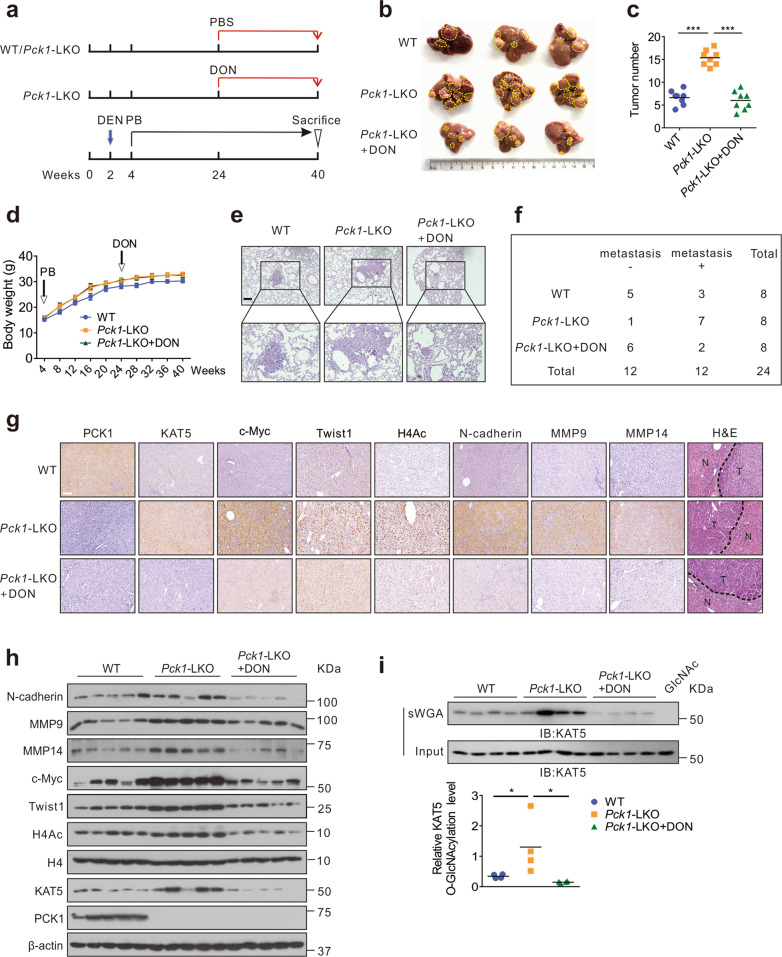
PCK1 deficiency promotes lung metastasis of HCC in vivo. **a** Schematics showing experimental design for procedures involving WT and *Pck1*-LKO mice. DEN diethylnitrosamine, PB phenobarbital, DON 6-diazo-5-oxo-L-norleucine. **b**, **c** Gross appearances of liver with tumors (**b**), and numbers of tumor nodules (**c**). *n* = 8 per group. The yellow dotted-line circles represent tumors. Data was shown as mean ± SD. One-way ANOVA followed by the Tukey test, ***P < 0.001. **d** The body weight of WT and *Pck1*-LKO mice injected with PBS or 1 mg/kg DON from week 24 to week 40, *n* = 8. All values are mean ± SD. **e** H&E staining for sections of metastasized lung. Scale bar: 200 μm. **f** The number of mice with lung metastatic tumors was determined by observation under anatomical microscope. **g**, **h** The indicated proteins expression in liver tumors were evaluated by immunohistochemical assay (**g**) and western blotting (**h**). Scale bar: 100 μm. **i** Analysis of KAT5 O-GlcNAcylation in mouse liver tumors by the sWGA pull-down assay. Statistical analysis was shown as mean ± SD (*n* = 4). One-way ANOVA followed by the Tukey test, *P < 0.05.

To further identify how PCK1 mediates KAT5 O-GlcNAcylation in HCC metastasis in vivo, WT and liver-specific *Pck1*-deletion (*Pck1*-LKO) mice were treated with 75 mg/kg of diethylnitrosamine (DEN) and fed with phenobarbital (PB) in a 0.05% diet to induce lung metastatic model of liver cancer [37]. One group of *Pck1*-LKO mice were administered an intraperitoneal injection of DON twice a week for 16 weeks (Fig. 6a). *Pck1*-LKO mice exhibited increased tumor sizes and number of tumor nodules, and DON treatment decelerated liver tumorigenesis without changing body weight (Fig. 6b–d). H&E staining for sections indicated that more *Pck1*-LKO mice had metastatic tumor nodules and larger size of tumor nodules in lung (Fig. 6e, f). The IHC and immunoblotting assays indicated that EMT-related molecules such as Twist1, c-Myc, N-cadherin, MMP9, MMP14 were increased in *Pck1*-LKO mice. In contrast, DON treatment partially alleviated the effects mediated by PCK1 deficiency (Fig. 6g, h). Consistent with the results in vitro, KAT5 O-GlcNAcylation levels were remarkably increased in *Pck1*-LKO mice (Fig. 6i). Collectively, these data demonstrate that PCK1 deficiency increases susceptibility to DEN/PB-induced lung metastasis of HCC by promoting O-GlcNAcylation of KAT5 in vivo.

### PCK1 downregulates KAT5 O-GlcNAcylation in human HCC

**Fig. 7:**
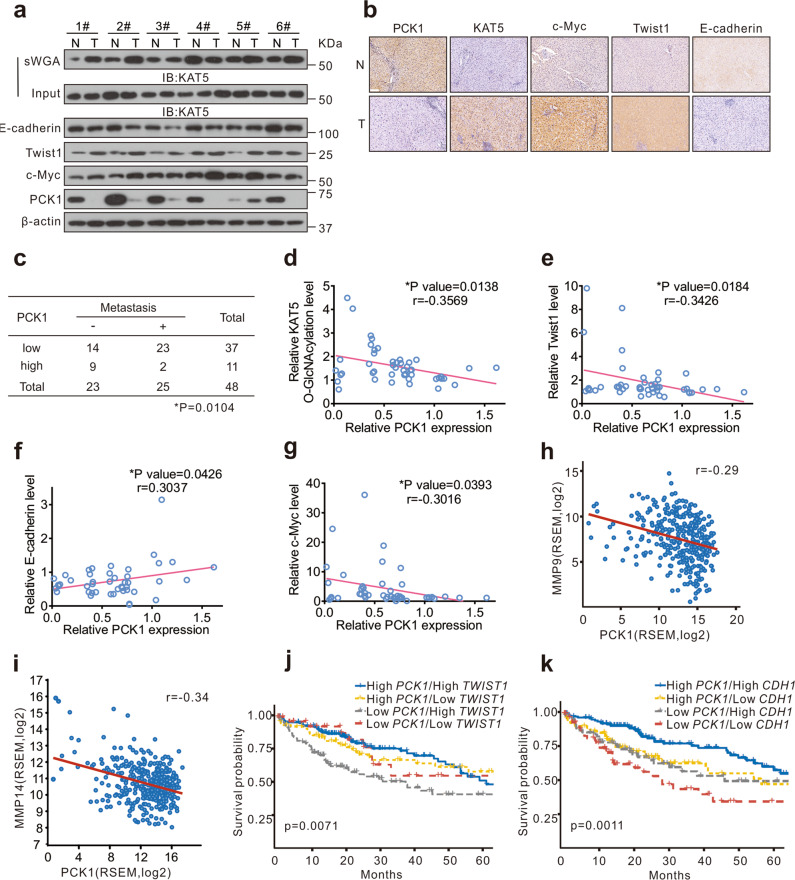
PCK1 expression negatively correlates with KAT5 O-GlcNAcylation and EMT-related markers in HCC tissues. **a**, **b** Immunoblots (**a**) or Immunohistochemistry images (**b**) of the indicated proteins in human HCC tissues and adjacent non-tumor tissues. Scale bar: 100 μm. **c** Correlation analysis of PCK1 expression and tumor metastasis in 48 patients with HCC. The relative PCK1 expression was normalized to β-actin, and classified into two levels (low, T < N; high, T > N). The data was analyzed using a *χ*^2^ test. *P < 0.05. **d**–**g** Correlation analysis of PCK1 expression and KAT5 O-GlcNAcylation level (**d**), PCK1 and Twist1 expression (**e**), PCK1 and E-cadherin expression (**f**), PCK1 and c-Myc expression (**g**) in 48 patients with HCC. Statistical analysis was performed using Pearson’s correlation coefficient (*r*). *P < 0.05. **h**, **i** Correlation analysis between *PCK1* and *MMP9* (**h**) or *MMP14* (**i**) mRNA expression levels based on data from 372 patients with HCC from The Cancer Genome Atlas (TCGA) database. Statistical analysis was performed using Pearson’s correlation coefficient (*r*). **j**, **k** The Kaplan–Meier survival curves depicting the overall survival (OS) of 372 patients with HCC obtained from the TCGA cohort.

Finally, we assessed PCK1, c-Myc, Twist1, E-cadherin expression and KAT5 O-GlcNAcylation in 48 paired human HCC tissues and tumor-adjacent tissues. The levels of PCK1 and E-cadherin were downregulated, and O-GlcNAcylation of KAT5, c-Myc, and Twist1 were upregulated in most HCC tissues (Fig. 7a, b and Supplementary Fig. 5a). Furthermore, we observed that tumor metastasis rate was inversely proportional to the expression of PCK1 in HCC (Fig. 7c and Supplementary Fig. 5a). In addition, KAT5 O-GlcNAcylation was negatively correlated with the expression level of PCK1 (Fig. 7d; Pearson correlation coefficient (*r*) = −0.3569). Consistent with our data in vitro, we found negative correlations between Twist1 and PCK1 expression (Fig. 7e; *r* = −0.3426), c-Myc and PCK1 expression (Fig. 7g; *r* = −0.3016), and positive correlations between PCK1 and E-cadherin (Fig. 7f; *r* = 0.3037). In an independent cohort of 372 HCC tissues from The Cancer Genome Atlas Liver Hepatocellular Carcinoma (TCGA-LIHC) dataset, *PCK1* was negatively correlated with *MMP9* or *MMP14* mRNA expression level (Fig. 7h, i; *r* = −0.29 (*MMP9*), −0.34 (*MMP14*)) and survival analysis showed that patients with lower levels of *PCK1* and higher levels of *TWIST1*, or lower levels of both *PCK1* and *CDH1* had poorer overall survival (OS) (Fig. 7j, k). In summary, the clinical validation supported the findings that PCK1 suppresses KAT5 O-GlcNAcylation and inhibits tumor metastasis in human primary HCC.

## Discussion

**Fig. 8:**
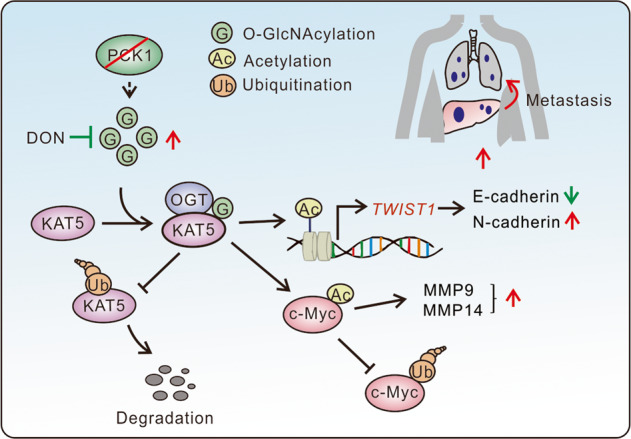
Schematic working model of PCK1 deficiency-mediated HCC metastasis. Gluconeogenesis enzyme PCK1 depletion promotes OGT-mediated O-GlcNAcylation of KAT5, thereby inhibiting its ubiquitination and degradation. Stabilized KAT5 promotes HCC metastasis through inducing the expression of EMT-related genes. DON 6-diazo-5-oxo-L-norleucine.

Metabolic reprogramming as hallmark of cancer is an emerging topic of study. Enhanced aerobic glycolysis of the tumor cells aggravates acidosis and immunosuppression, and leads to invasion and metastasis of HCC [38, 39]. Several studies have reported that gluconeogenesis play tumor-suppressing roles in HCC by antagonizing glycolysis [5, 8–11, 40]. In addition, a recent report showed that PCK1 drove liver metastasis of colorectal cancer by promoting nucleotide synthesis under hypoxia [41]. Our previous study demonstrated that PCK1 plays a tumor suppressor role in HCC via decreasing UDP-GlcNAc biosynthesis and global O-GlcNAcylation [16]. However, the role of PCK1 in HCC metastasis remains unclear. In this study, we first revealed that PCK1 inhibits *TWIST1* transcription, acetylation of c-Myc, and EMT in HCC by O-GlcNAcylation of KAT5 (Fig. 8). This study uncovered a link for gluconeogenesis disruption, O-GlcNAcylation modification and epigenetic regulation in HCC metastasis.

PTM of proteins is vital for various cellular processes. O-GlcNAcylation is the dynamic PTM that regulates the biological function of protein upon nutrient demand [42]. However, no evidence has been found regarding O-GlcNAcylation of the MYST family. KAT5 is an acetyltransferase that belongs to MYST family and plays essential roles in many cellular events including chromatin remodeling, DNA damage response, gene transcriptional regulation, cGAS-mediated innate antiviral response, autophagy, and pathological processes, such as tumorigenesis and metastasis [22, 43–45]. KAT5 was involved in HCC tumor cell growth and promoted EMT by acetylation of the SPZ1-TWIST1 complex [46, 47]. Furthermore, several PTMs, including phosphorylation, SUMOylation, and ubiquitination have been reported to be critical for KAT5 function [29, 30, 48, 49]. Phosphorylation of KAT5 at different sites has different regulatory effects on KAT5. For instance, S90 phosphorylation was associated with the transcription machinery, but S86 phosphorylation by GSK3 regulated HAT activity of KAT5 [48, 49]. Moreover, SUMOylation of KAT5 at Lys430 attenuated its interaction with DNA-PKcs in S-phase cells [29]. Herein, we identified, by in vitro functional analysis, that Ser119 is a O-GlcNAcylation site on KAT5. Importantly, we found that O-GlcNAcylation increased KAT5 stability, thereby inducing epigenetic activation of *TWIST1*, associated with the acquisition of a metastatic phenotype in PCK1-deficient hepatoma cells. Interestingly, S119A mutant did not completely destroy the O-GlcNAcylation of KAT5, suggesting the possibility that beside S119, other O-GlcNAcylation target sites may exist. Further studies are needed to explore the potential of O-GlcNAcylation sites contributing to post-translational activation of KAT5 by mass spectrum identification.

In the absence of stimuli, cellular KAT5 protein is maintained at a low level via proteasomal degradation. KAT5 is targeted for degradation through mono- and polyubiquitination by mouse double minute 2 (Mdm2) [50], and a Mdm2-linked, Tat-dependent, CBP/p300-associated E4 ubiquitin ligase [30]. Meanwhile, DNA damaging signals, such as UV, inhibit Mdm2-driven ubiquitination and KAT5 levels rapidly stabilize to participate in the DNA damage response [50]. Here, we found that KAT5 maintained low expression level in PLC/PRF/5 cells with high expression of PCK1. PCK1 weakened the stability of KAT5, accelerated its ubiquitination and degradation by decreasing KAT5 O-GlcNAcylation, leading to an imbalance between protein synthesis and degradation, thereby accelerating protein turnover. PCK1 regulates KAT5 ubiquitination and stability by triggering a post-translational modification cascade involving O-GlcNAcylation and ubiquitination. Further studies are needed to explore other proteins regulated by PCK1 through this post-translational modification cascade. And other functions of this cascade, such as signal transduction, DNA damage repair, endocytic pathway, should be investigated in the future.

KAT5, as an acetyltransferase, has been reported mostly as a transcriptional regulator. It also acts as a key regulator by targeting non-histone proteins including p53-mediated apoptosis, ATM-activated DNA damage responses, UBF-regulated ribosomal gene transcription, and upregulating transcriptional repressor activity of FOXP3 in primary human regulatory T cells [24, 51–53]. Previous studies have reported that KAT5 acetylates c-Myc, thereby increasing its stability and promoting tumor metastasis [36]. The transcription factor c-Myc, as one of the most frequently overexpressed oncogenes in cancers, plays a pivotal role in metastasis [54]. As for regulation of c-Myc, our data showed that O-GlcNAcylation of KAT5 attenuated the degradation of c-Myc protein without affecting its mRNA level, suggesting that KAT5 regulates c-Myc at the post-transcriptional level. Importantly, O-GlcNAcylation of KAT5 enhanced interaction between KAT5 and c-Myc, and increased acetylation of c-Myc, thus highlighting the importance of O-GlcNAc modification in tightly regulating the interaction between KAT5 and other non-histone binding partners. Other PTMs on KAT5, such as phosphorylation and SUMOylation need to be further explored for possible crosstalk with O-GlcNAcylation.

TWIST, one of the EMT-inducing transcription factors, inhibits the expression of markers associated with the epithelial state, such as E-cadherin, and concomitantly activates the expression of molecules associated with the mesenchymal state, including N-cadherin [12]. Recent studies have reported that TWIST can be acetylated by KAT5 in cancer metastasis [27, 47]. In our study, the data showed that the mRNA level of *TWIST1* was upregulated in PCK1-deficient hepatoma cells, suggesting that PCK1 may transcriptionally inhibit *TWIST1* by O-GlcNAcylation of KAT5. Further studies are needed to verify whether TWIST1 can be acetylated by KAT5 in PCK1-deficient hepatoma cells. On the other hand, epigenetic modifications, including histone methylation and acetylation, play important roles in the transcriptional activation of *TWIST1* expression. Previous findings showed that both histone methyltransferase MMSET, Frizzled family receptor 7 (FZD7), and acetyltransferase CBP regulate the epigenetic modifications of histone H3 on the *TWIST1* promoter [55–57]. Herein, we first found that apart from epigenetic modifications of histone H3, acetylation of H4 also plays vital roles in *TWIST1* transcription. Importantly, we uncovered a novel mechanism that O-GlcNAcylation of KAT5, as a transcriptional regulator, epigenetically activates *TWIST1* via enhancing histone H4 acetylation on the *TWIST1* promoter, thereby inhibiting E-cadherin expression in PCK1-deficient hepatoma cells. Indeed, the correlations among PCK1, KAT5, TWIST1, and E-cadherin can be observed in human HCC tissues.

In summary, we demonstrated that the OGT-mediated O-GlcNAcylation of KAT5 increases the stability of KAT5. Moreover, PCK1 depletion promotes HCC metastasis via epigenetic activation of *TWIST1* and acetylation of c-Myc by O-GlcNAcylation of KAT5. These results revealed that O-GlcNAcylation of KAT5 plays double roles in PCK1 deficiency-inducing HCC metastasis, including transcriptional activation of *TWIST1* and acetylation of c-Myc. Furthermore, the study broadens our understanding of PCK1 in HCC progression and metastasis, and indicate that targeting O-GlcNAcylation has the potential to become a novel strategy for HCC therapy.

## Materials and methods

Full details are available in Supplementary material and methods.

### Clinical specimens

HCC tumor tissues and paired non-tumorous tissue samples were collected from 48 patients undergoing surgery at the first Affiliated Hospital of Chongqing Medical University. All patients provided an informed consent and had not received chemotherapy or radiation therapy before surgery. This study was approved by the Institutional Ethical Review Board of Chongqing Medical University.

### Cell cultures and reagents

PLC/PRF/5, SK-Hep1 were obtained from the American Type Culture Collection (Manassas, VA), MHCC-97H was from the Cell Bank of the Chinese Academy of Sciences (Shanghai, China). All cell lines were confirmed free of mycoplasma (MycoAlert PLUS kit; Lonza, Basel, Switzerland) and cell authentication was performed by short tandem repeat profiling (Beijing Microread Gene Technology Co., Beijing, China). Cells were cultured in Dulbecco’s modified Eagle’s medium (DMEM; Gibco, Grand Island, NY, USA) supplemented with 10% FBS (Corning, NY, USA), 100 mg/mL streptomycin, and 100 IU penicillin at 37 °C containing 5% CO_2_.

Cells were cultured in medium supplemented with 6-diazo-5-oxo-l-norleucine (DON, 20 μM; D2141; Sigma-Aldrich, St Louis, MO, USA), ST045849 (ST, 50 μM; MFCD03308174; Tim Tec, Newark, USA), Thiamet G (TG, 25 μM; S7213; Selleckchem, Houston, USA) or Cycloheximide (CHX, 100 μM; HY-12320; MedChemExpress, New Jersey, USA) and then collected for analysis.

### Adenovirus production and construction of stable cell lines

Recombinant adenovirus, AdPCK1 and AdG309R, were produced using the AdEasy system as previously described [11]. AdGFP was used as a negative control (kindly provided by Dr. Tong-Chuan He, University of Chicago, USA).

The CRISPR-Cas9 system was kindly gifted from Prof. Ding Xue (the School of Life Sciences, Tsinghua University, Beijing, China). PCK1- and KAT5-knockout cells were established as previously described [11]. The knockout efficiency was validated by immunoblotting. Sequences of sgRNAs are listed in Supplementary Table 1.

### Lentivirus-mediated RNA interference

To knock down OGA or OGT expressing, four pairs of oligonucleotides encoding shRNA were designed and cloned into the lentiviral vector pLL3.7 (kindly provided by Prof. Bing Sun from Center for Excellence in Molecular Cell Science, CAS, China). A negative control construct (shCon) was also generated. The lentiviral supernatants were produced in HEK-293T cells as previously described [11]. Primer sequences are listed in Supplementary Table 1.

### Immunoprecipitation assay

Cells were lysed with lysis buffer (50 mM Tris-HCl, pH 7.4, 1 mM EDTA, 150 mM NaCl, and 1% Triton X-100) containing 1× Protease Inhibitor Cocktail (Roche, Indianapolis, IN) and 1× Phosphatase Inhibitor (Beyotime). Supernatants were separated and incubated with anti-FLAG (F3165; Sigma), anti- OGT (ab96718, Abcam), anti-KAT5 (sc166323; Santa), or anti-c-Myc (10828-1-AP; Proteintech Group Inc.) overnight at 4 °C. Protein-antibody complexes were incubated with protein A/G agarose beads (Millipore) for 4 h. The complexes were eluted and resolved to immunoblotting with the indicated antibodies. The horseradish peroxidase-conjugated secondary antibody was goat anti-mouse IgG (ab6789; Abcam) or mouse anti-rabbit IgG, light chain specific (211-032-171; Jackson ImmunoResearch, Lancaster, USA).

### sWGA pull-down assay

Liver tissues or hepatic cells were lysed in Lysis 125 buffer (50 mM Tris, pH 7.4, 125 mM NaCl, 5 mM EDTA, 5 mM EGTA, 0.1% Nonidet P-40, 50 mM NaF, 1 mM PMSF, and 1× Proteinase Inhibitor Cocktail (Roche)). The supernatant was denatured in glycoprotein denaturing buffer and digested with PNGase (P0704S; New England Biolabs, USA) to remove N-linked glycoproteins. Pre-cleared supernatant was incubated with succinylated wheat-germ agglutinin (sWGA)-conjugated agarose beads (Vector Laboratories, Burlingame, CA) overnight at 4 °C. Precipitated complexes were eluted and immunoblotted with anti-KAT5 antibodies.

### Chromatin immunoprecipitationm assay

In all, 6 × 10^6^ cells were cross-linked using 1% paraformaldehyde for 10 min at 37 °C. Cell lysates were sonicated by the Bioruptor at high-output power for 15 cycles (30 s ON and 30 s OFF). Supernatants were separated and incubated with anti-acetylated H4 (17-630, Sigma) or control IgG overnight at 4 °C. Chromatin-antibody complexes were collected by protein A/G agarose beads (Millipore), washed and then eluted. DNA complexes were reverse cross-linked in a water bath at 65 °C for 4 h and treated with proteinase K. DNAs were purified with phenol-chloroform-isopentanol, and ethanol precipitated. and then quantified by real-time PCR. Primers are listed in Supplementary Table 1.

### Animal models

For the orthotopic lung metastasis model, BALB/c nude mice (5–6 weeks of age) were randomly divided into 4 groups (*n* = 6/group). MHCC-97H cells were non-infected or infected with AdGFP, AdPCK1, or AdG309R mutant for 24 h, then collected and injected into the left lobe of nude mice livers (1 × 10^5^ cells/ injection). The animals were sacrificed 7 weeks after the injections and lung tissues were collected for histological examination.

To generate *AlbCre*^(+/−)^, *Pck1*^(flox/flox)^ (liver-specific knockout, *Pck1*-LKO) mice, *AlbCre*^*(+/−)*^ mice (purchased from Model Animal Research Center of Nanjing University, Nanjing, China) were crossed with *Pck1*^(flox/flox)^ mice on the 129 background (from the Mutant Mouse Resource & Research Centers, MMRRC:011950-UNC) as previously described [58], and *AlbCre*^(−/−)^, *Pck1*^(flox/flox)^ (wild-type, WT) mice were referred to as a control (*n* = 8/group). For the spontaneous lung metastasis model of hepatocellular carcinoma, a dose of 75 mg/kg diethylnitrosamine (DEN; Sigma) was injected into 2-week-old mice. Mice were fed with phenobarbital in a 0.05% diet from week 4 to the final sacrifice [37]. At 24 weeks, the *Pck1-*LKO mice were intraperitoneally injected with 1 mg/kg DON twice per week for 16 weeks. At 40 weeks, mice were sacrificed, and lung and liver tissues were collected for examination. All animal procedures were approved by the Research Ethics Committee of Chongqing Medical University (reference number: 2017012).

### TCGA data analysis

Gene expression data and corresponding clinical data for 372 patients with HCC were obtained from The Cancer Genome Atlas Liver Hepatocellular Carcinoma (TCGA-LIHC) dataset [59]. The Kaplan–Meier survival curves were generated by ‘survminer’ package of R (Version 3.6.3).

### Statistical analysis

Data were presented as the mean ± SD. Tests used to examine the differences between groups include Student’s *t* test, one-way ANOVA and *χ*^2^ test. Pearson correlation coefficient (*r*) was used to test the linear correlation. *P*-values < 0.05 were considered statistically significant. *P < 0.05, **P < 0.01, ***P < 0.001. Statistical analyses were conducted using GraphPad Prism 6.0 software (La Jolla, CA, USA).

## Supplementary information

Supplementary materials and methods

Supplementary figure legends

Supplementary Table 1

Supplementary Figure 1

Supplementary Figure 2

Supplementary Figure 3

Supplementary Figure 4

Supplementary Figure 5
