# Supplementary material for: O-GlcNAc modified-TIP60/KAT5 is required for PCK1 deficiency-induced HCC metastasis
Source: Oncogene. 2021 Oct 14;40(50):6707–19. doi: 10.1038/s41388-021-02058-z (PMC8677624; doi:10.1038/s41388-021-02058-z)
Supplement: Supplementary file 1 — Supplementary materials and methods [file 41388_2021_2058_MOESM1_ESM.docx]

**Supplementary materials & methods**

**Plasmid constructs**

The full-length cDNA of human *Tip60/KAT5* (NM_182710.3) and S119A, T197A, T246A mutants, the truncations mutants of KAT5 containing Chromo (1-209aa), Zn finger (76-315aa), HAT (233-513aa) domains were constructed into pAdTrack-TO4-GFP-2Flag vector, respectively. The full-length cDNA of human *PCK1* (NM_002591), G309R mutant (PCK1 enzyme inactive), *KAT5* (NM_182710.3) and *OGT* (NM_181672.2) were constructed into pBudCE4.1-3HA vector, respectively. Primers are listed in Supplementary Table 1.

**Quantitative real-time PCR**

Total RNA was extracted from human hepatoma cells using TRIzol reagent (Invitrogen, Rockville, MD, USA). RNA was reverse transcribed into cDNA using PrimeScript™ RT Reagent Kit (RR047A, TaKaRa, Tokoyo, Japan). Quantitative real-time PCR was performed using Bio-Rad CFX96 machine (Bio-Rad, Hercules, CA, USA). The mRNA levels were calculated by the 2^–ΔΔCt^ method using β-actin as the normalization control. Primer sequences are listed in Supplementary Table 1.

**Wound-healing assay**

Cells were cultured in 96-well plates, and wounds were created using WoundMaker™ on the cell surface. After 24h (SK-Hep1 cell line) or 36h (PLC/PRF/5 cell line), the wound areas were recorded by the IncuCyte ZOOM Live-Cell Imaging system (Essen BioScience, Ann Arbor, MI, USA).

**Transwell migration assay**

Cell invasion was assessed using Cell Culture Insert (FALCON, USA). Cells were replete onto the upper chamber with serum-free medium. The lower chamber was replete with medium containing 10%FBS. The invasive cells were stained with crystal violet and quantified (3 random 200× fields per well) under Axio Imager A2 (ZEISS, Germany).

**Western blotting**

Proteins were collected from cells or tissues by lysis buffer (Beyotime, Shanghai, China) containing 1 mM phenylmethylsulfonyl fluoride. Proteins were separated by SDS/PAGE and electro-transferred to PVDF membranes (Millipore, Billerica, MA, USA). The membranes were incubated with primary antibodies against PCK1 (1:1000; BS6870; Bioworld, Atlanta, GA, USA), E-cadherin (1:10000; ab40772; Abcam, Cambridge, UK), N-cadherin (1:1000; 13116; Cell Signaling Technology, Danvers, MA, USA), O-GlcNAc (1:1000; ab2739; Abcam), OGT (1:2000; ab96718; Abcam), OGA (1:5000; ab124807; Abcam), FLAG(1:2000; F3165; Sigma-Aldrich), HA (1:2000; 26183; Invitrogen, Carlsbad, CA, USA), KAT5 (1:2000; ab23886; Abcam), c-Myc (1:2000; 10828-1-AP; Proteintech Group Inc., Rosemont, IL, USA;),Twist1(1:1000; 25465-1-AP; Proteintech Group Inc.), MMP9 (1:1000; BS1241; Bioworld), MMP14 (1:1000; BS9899M; Bioworld), Acetylated lysine (1:1000; 9441; Cell Signaling Technology), H3 (1:5000; H0164; Millipore), H4 (1:5000; 07-108; Millipore) , acetyl-Histone H3 (1:1000; 06-599; Millipore), acetyl-Histone H4 (1:1000; 06-598; Millipore), KAT1 (1:1000; ab194296; Abcam), KAT2A (1:1000; ab217876; Abcam), KAT2B (1:2000; ab176316; Abcam), KAT3B (1:1000; ab275378; Abcam), KAT7 (1:5000; ab190908; Abcam), KAT8 (1:1000; ab200660; Abcam). Then, the membranes were incubated with horseradish peroxidase-conjugated secondary antibody (Abcam, Cambridge, UK). β-actin (TA-09; ZSGB-BIO, Bejing, China) was used as a normalization control.

**KAT5 protein stability assay**

For stability detection of endogenous KAT5 protein, MHCC-97H cells were treated with 100 μM cycloheximide (CHX) and collected at 0, 2, 4 and 6 h. Cells were lysed and KAT5 was detected by an anti-KAT5 antibody (ab23886, Abcam). For protein stability detection of KAT5 WT and S119A mutant, PCK1-OE MHCC-97H cells or PCK1-KO cells were transfected with Flag-KAT5 or Flag-S119A mutant plasmids and treated with 100 μM CHX and harvested at 0, 2, 4 and 6 h. Cells were lysed and KAT5 was detected using an anti-FLAG antibody (F3165, Sigma). OGT was regulated by selective inhibitor ST045849 or Thiamet G (O-GlcNAcase inhibitor).

**Immunohistological (IHC) staining**

Tissues were fixed in fresh 4% paraformaldehyde and embedded in paraffin. Sections were incubated with primary antibodies against PCK1 (1:500), KAT5 (1:500), c-Myc (1:100), N-cadherin (1:100), E-cadherin (1:500), MMP9 (1:100), MMP14 (1:100), Twist1 (1:200) or acetyl-Histone H4 (1:200) overnight at 4°C. The sections were incubated with a secondary anti-rabbit IgG (ZSGB-BIO) and stained using 3,3′-diaminobenzidine (ZSGB-BIO). Stained slides were scanned with a Pannoramic Scan 250 Flash and images were acquired using Pannoramic Viewer 1.15.2 (3DHistech, Budapest, Hungary).

**Immunofluorescence staining**

KAT5 and OGT were detected with anti-KAT5 (1:100; sc166323; Santa Cruz Biotechnology, CA, USA), anti- OGT (1:200; ab96718, Abcam) in MHCC-97H cells, and specific signals were visualized with Alexa Fluor 488 or 552 secondary antibody (Invitrogen). Nucleus was stained using 1μg/ml DAPI (10236276001, Roche Diagnostics GmbH, Mannheim, Germany). Stained sections were observed by a laser-scanning confocal microscope (Leica TCS SP8, Solms, Germany).

**Ubiquitination assay**

Cells were lysed in SDS lysis buffer (50 mM Tris-HCl, pH 7.5; 150 mM NaCl; 1% SDS) containing 1× Protease Inhibitor Cocktail (Roche) and 1× Phosphatase Inhibitor (Beyotime). Supernatants were separated and incubated with an anti-FLAG (F3165; Sigma), anti-KAT5 (sc166323; Santa), or anti-c-Myc (10828-1-AP; Proteintech Group Inc.) overnight at 4 °C. After 4 h incubation with protein A/G agarose beads (Millipore), protein-antibody complexes were eluted and immunoblotted using the indicated antibodies.

**Prediction of O-GlcNAcylation sites**

O-GlcNAcylation sites of KAT5 were predicted using the online database (YinOYang 1.2 Server), as previously described^1^.

**References**

1 Gupta R. Prediction of glycosylation across the human proteome and the correlation to protein function. *Pac Symp Biocomput* 2002; **7**: 310–322.
