# Supplementary material for: O-GlcNAc modified-TIP60/KAT5 is required for PCK1 deficiency-induced HCC metastasis
Source: Oncogene. 2021 Oct 14;40(50):6707–19. doi: 10.1038/s41388-021-02058-z (PMC8677624; doi:10.1038/s41388-021-02058-z)
Supplement: Supplementary file 2 — Supplementary figure legends [file 41388_2021_2058_MOESM2_ESM.docx]

**Supplementary figure legends**

**Supplementary Figure 1.** Inhibition of O-GlcNAcylation reverses the detrimental effects of PCK1 on hepatoma cell invasion and migration.

**a-b** Global O-GlcNAcylation levels in parental or PCK1-KO cells **(a)** or in MHCC-97H cells infected with AdGFP, AdPCK1 or AdG309R mutant **(b)** were detected by immunoblotting. **c-j** Representative and quantified results of the transwell **(c.e.g.i)** and wound-healing assays **(d.f.h.j)** in PCK1-KO cells treated with 50 μM ST045849 (ST) **(c.d)** or transduced with shOGT or shCon **(e.f)** or in PCK1-OE SK-Hep1 cells treated with 25 μM Thiamet G (TG) **(g.h)** or transduced with shOGA or shCon **(i.j)**. Statistical analysis was shown as mean ± SD (n=3). One-way ANOVA followed by the Tukey test, *P < 0.05, **P < 0.01, ***P < 0.001. Scale bar: 100 μm. **k-l** Interaction between exogenous HA-OGT and Flag-KAT5 in MHCC-97H cells. The cell lysates were immunoprecipitated with anti- FLAG or anti-HA antibody and detected with an anti-HA or anti- FLAG antibody.

**Supplementary Figure 2.** O-GlcNAcylation increases the stability of KAT5 by suppressing its ubiquitination.

**a** The half-life of exogenous Flag-KAT5 in MHCC-97H cells was measured by immunoblotting and quantitative analysis. Cells were treated with 25 μM Thiamet-G (TG) for 12 h, and protein synthesis was blocked by treatment with 100 μM cyclohexamide (CHX) for the indicated times. The levels of KAT5 were normalized to those of β-actin, and the 0 h points were arbitrarily set to 100%. Data are representative of at least 3 independent experiments. **b** Exogenous Flag-KAT5 ubiquitination in MHCC-97H cells. Cells were co-transfected with Flag-KAT5 and HA-ubiquitin for 48 h and treated with 25 μM TG for 12 h. Flag-KAT5 was immunoprecipitated using anti-Flag antibody. **(c-d)** Protein expression of KATs in PCK1-KO cells **(c)** or in SK-Hep1 cells infected with AdGFP, AdPCK1 or AdG309R mutant; mock is a blank control **(d)**. **(e-f)** The mRNA or protein expression levels of KAT5 in MHCC-97H cells infected with AdGFP, AdPCK1, AdG309R mutant were measured by qRT-PCR **(e)** or immunoblotting **(f)**. Statistical analysis was shown as indicated (n=3). All values are mean ± SD. Student’s t-test, ns, not significant.

**Supplementary Figure 3.** Loss of KAT5 retards PCK1 deficiency-promoted hepatoma cell migration and EMT process.

**a-b** Representative and quantified results of the transwell **(a)** and wound-healing assays **(b)** in KAT5-KO cells. Statistical analysis was shown as mean ± SD (n=3). One-way ANOVA followed by the Tukey test, **P < 0.01, ***P < 0.001. Scale bar: 100 μm. **c** Levels of H3Ac and H4Ac were measured by immunoblotting in MHCC-97H cells infected with AdGFP, AdPCK1, AdG309R mutant. **d** The mRNA expression levels of EMT-related transcription factors in MHCC-97H cells infected with AdGFP, AdPCK1 were measured by qRT-PCR. Statistical analysis was shown as mean ± SD (n=3). Student’s t-test, ns, not significant, *P < 0.05, **P < 0.01. **e-g** The protein expression levels of EMT-related markers were measured by immunoblotting in MHCC-97H cells infected with AdGFP, AdPCK1 or AdG309R mutant **(e)**, in PCK1-KO cells transduced with shOGT or shCon **(f)** or in MHCC-97H cells infected with AdGFP, AdPCK1 and transduced with shOGA or shCon **(g)**.

**Supplementary Figure 4.** Re-expression of KAT5 restores the invasion ability of KAT5-KO or PCK1-KO cells.

**a-b** The protein expression levels of EMT-related markers were measured by immunoblotting in PCK1-KO cells transduced with shOGT or shCon **(a)** or in MHCC-97H cells infected with AdGFP, AdPCK1 and transduced with shOGA or shCon **(b)**. **c-d** Representative and quantified results of the Transwell **(c)** and wound-healing assays **(d)** in KAT5-KO cells transfected with vector control, Flag-tagged KAT5 WT, or S119A mutant. Statistical analysis was shown as mean ± SD (n=3). One-way ANOVA followed by the Tukey test, *P < 0.05, **P < 0.01. Scale bar: 100 μm.

**Supplementary Figure 5.** PCK1 and O-GlcNAcylation of KAT5 expression in HCC tissues.

**a** Immunoblot of the indicated proteins in human HCC tissues and adjacent non-tumor tissues.
