# Supplementary figures and images for: O-GlcNAc modified-TIP60/KAT5 is required for PCK1 deficiency-induced HCC metastasis
Source: Oncogene. 2021 Oct 14;40(50):6707–19. doi: 10.1038/s41388-021-02058-z (PMC8677624; doi:10.1038/s41388-021-02058-z)

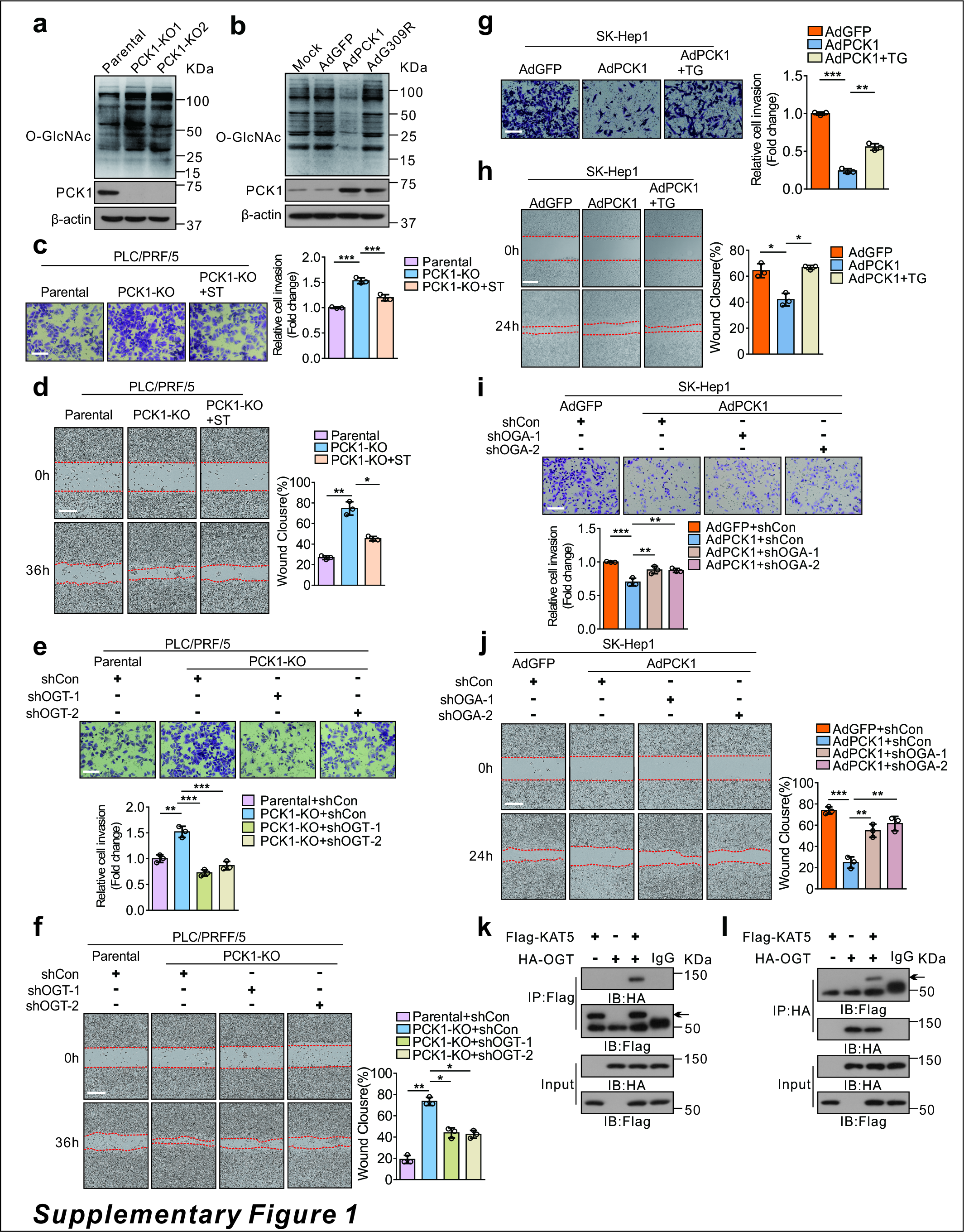

Supplement: Supplementary file 4 — Supplementary Figure 1 [file 41388_2021_2058_MOESM4_ESM.tif]

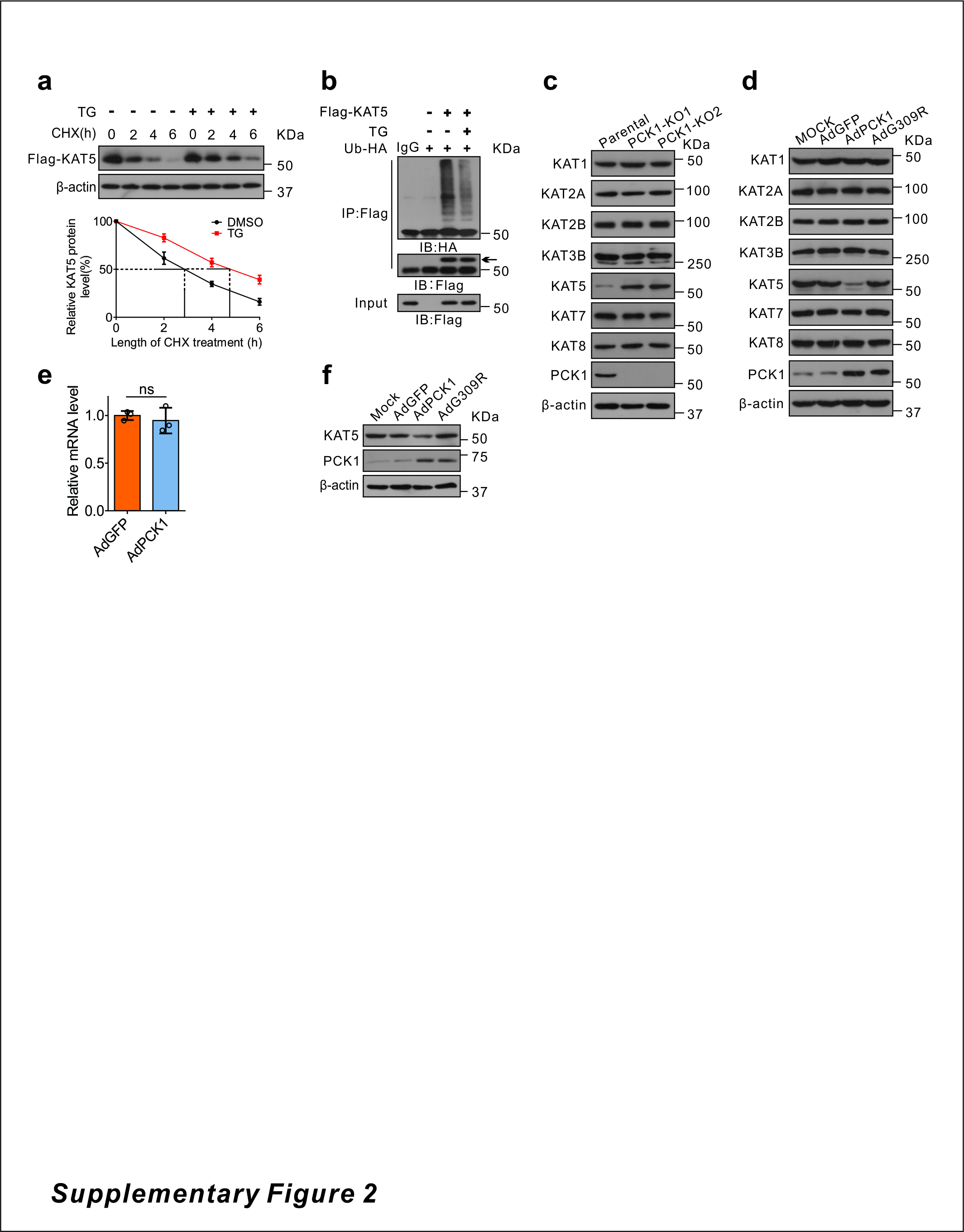

Supplement: Supplementary file 5 — Supplementary Figure 2 [file 41388_2021_2058_MOESM5_ESM.tif]

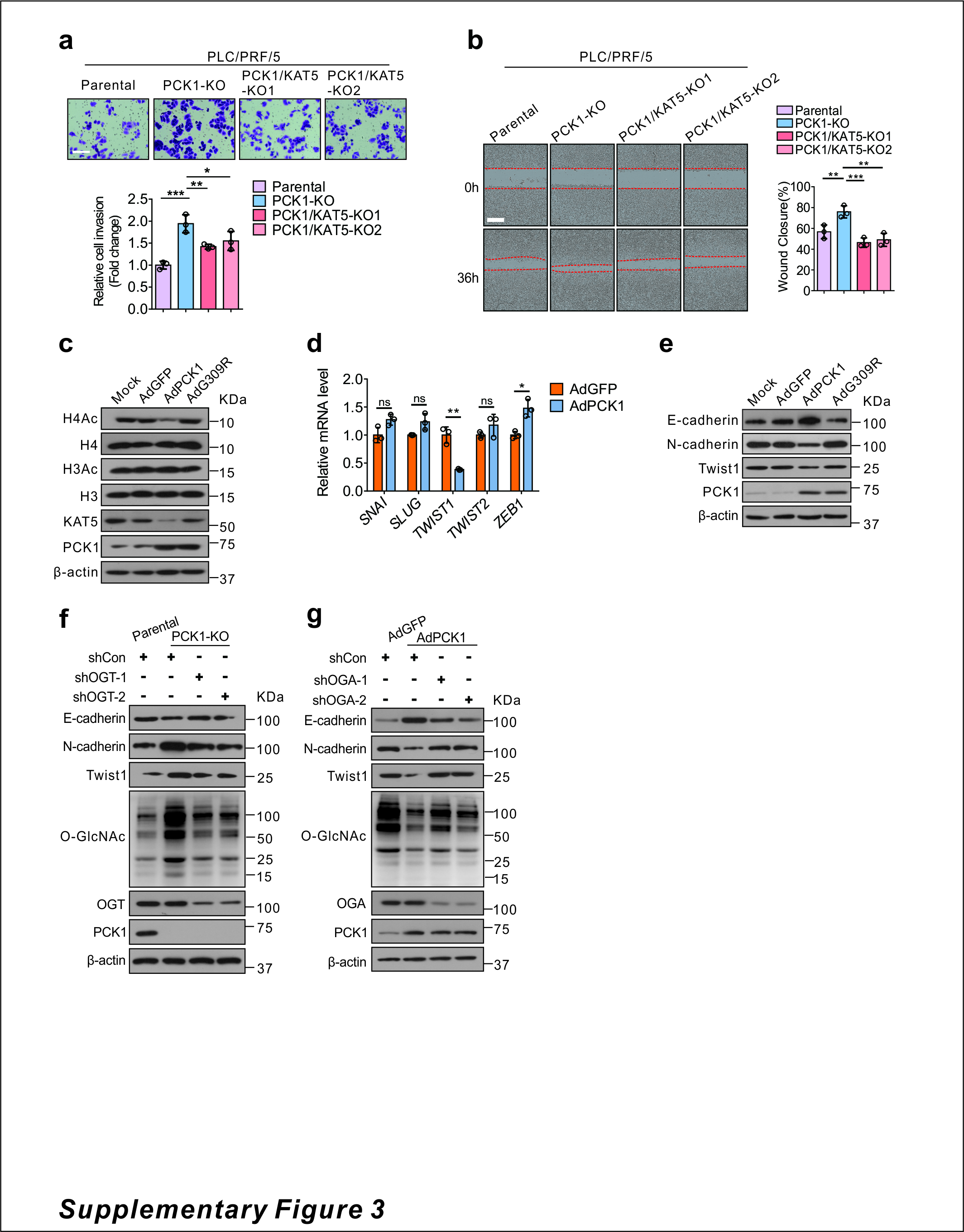

Supplement: Supplementary file 6 — Supplementary Figure 3 [file 41388_2021_2058_MOESM6_ESM.tif]

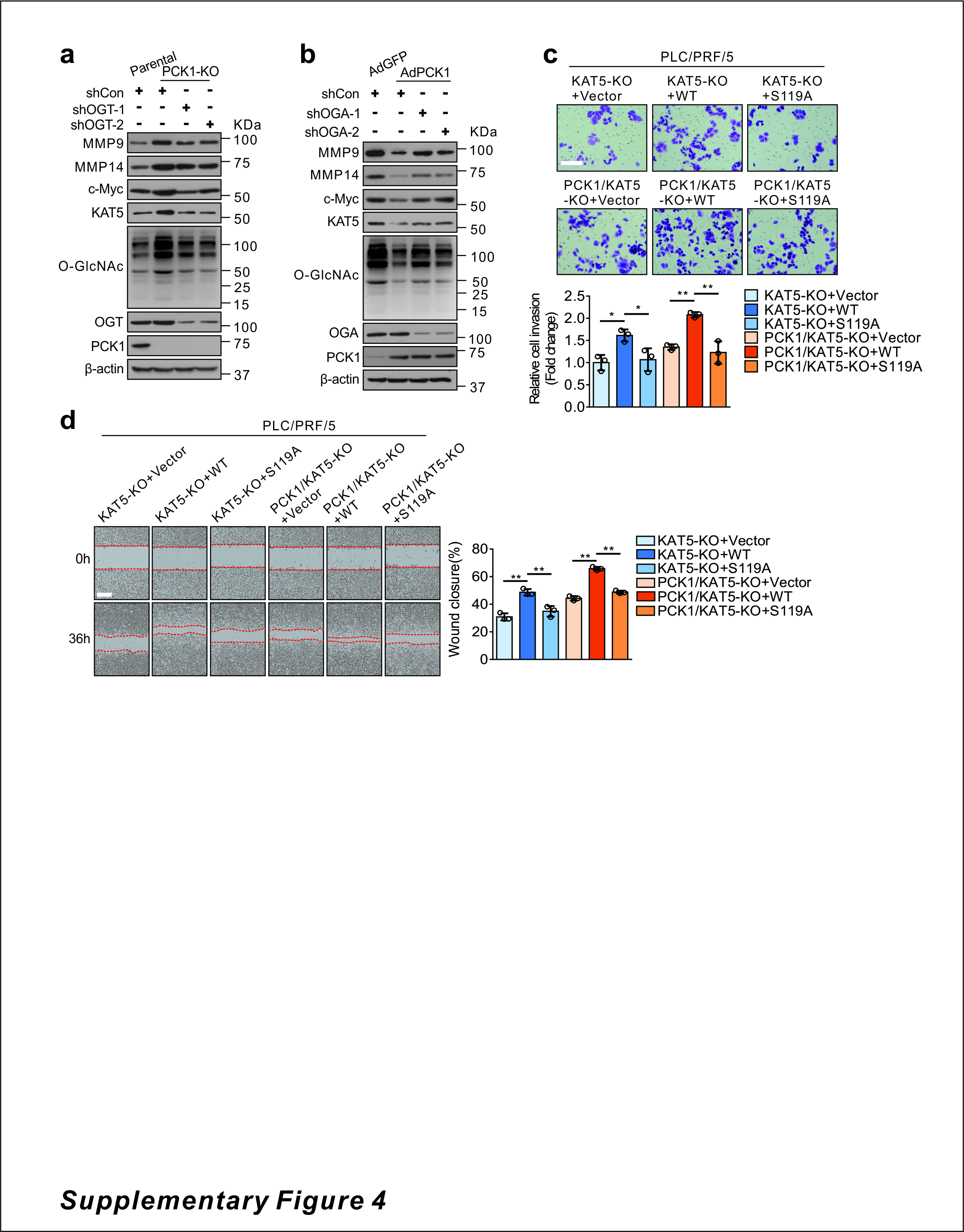

Supplement: Supplementary file 7 — Supplementary Figure 4 [file 41388_2021_2058_MOESM7_ESM.tif]

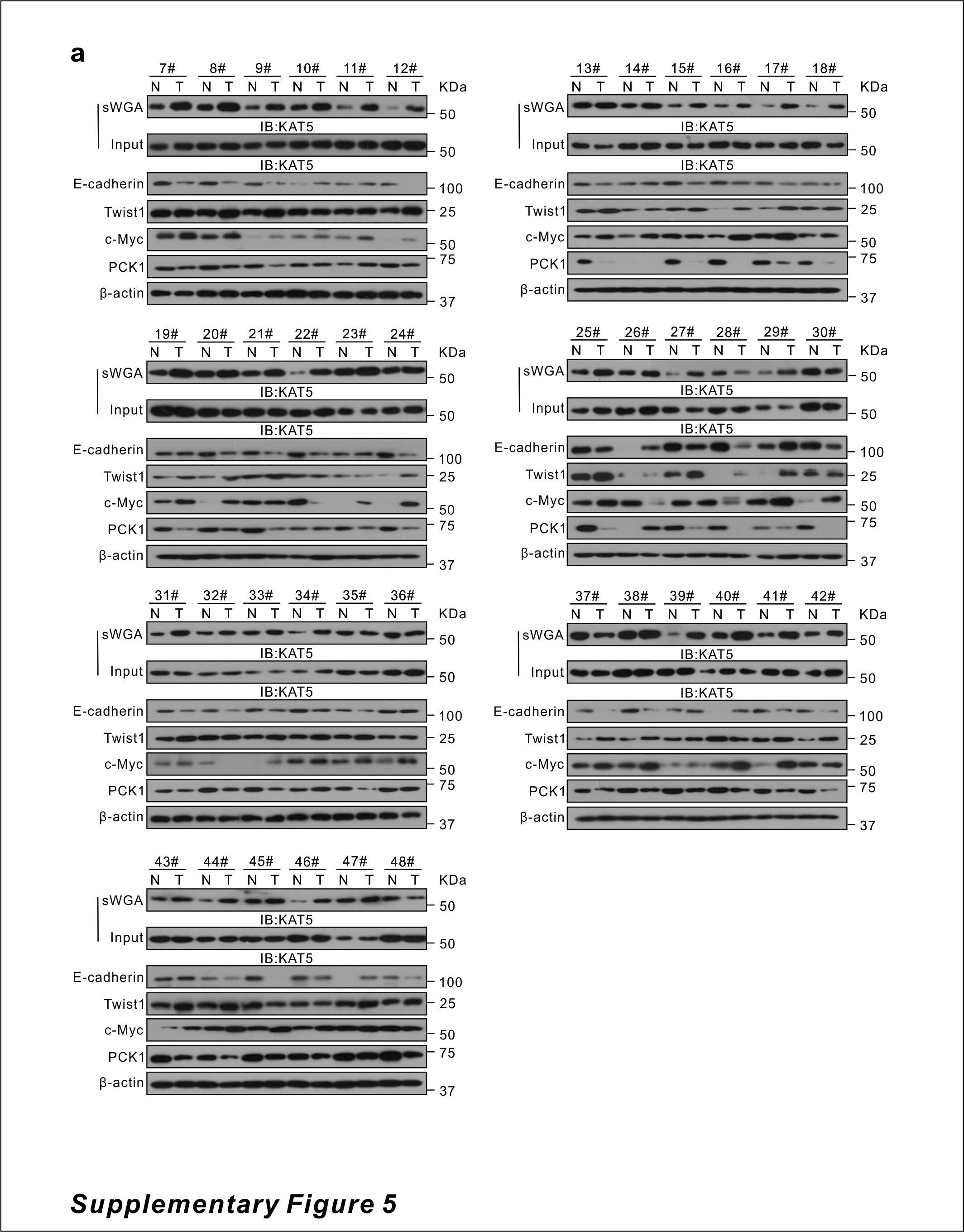

Supplement: Supplementary file 8 — Supplementary Figure 5 [file 41388_2021_2058_MOESM8_ESM.tif]
